# Supplementary material for: Sensitization of Guinea Pig Skin to Imported Fire Ant Alkaloids and Establishment of an Inflammatory Model
Source: Int J Environ Res Public Health. 2023 Jan 20;20(3):1904. doi: 10.3390/ijerph20031904 (PMC9914866; doi:10.3390/ijerph20031904)

Mass spectral data

1/alkaloid

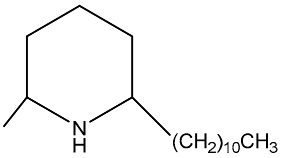

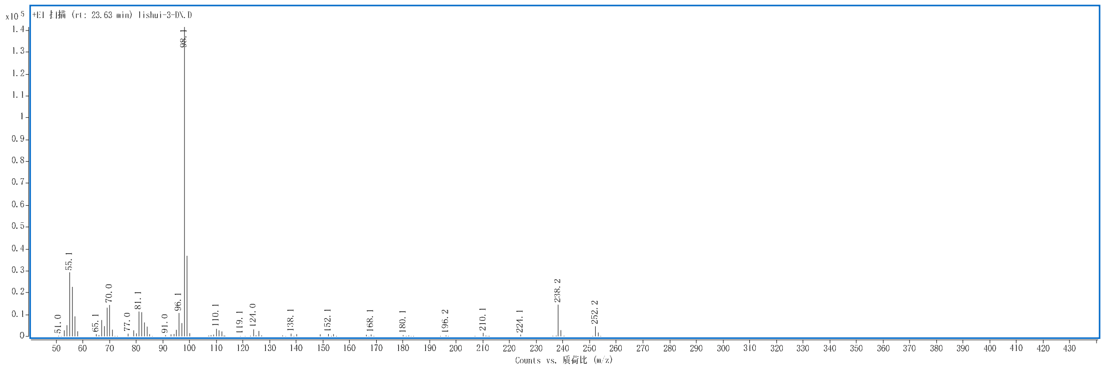

2/alkaloid

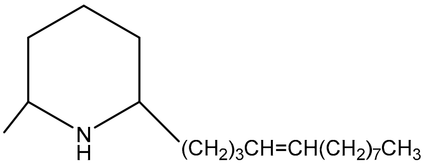

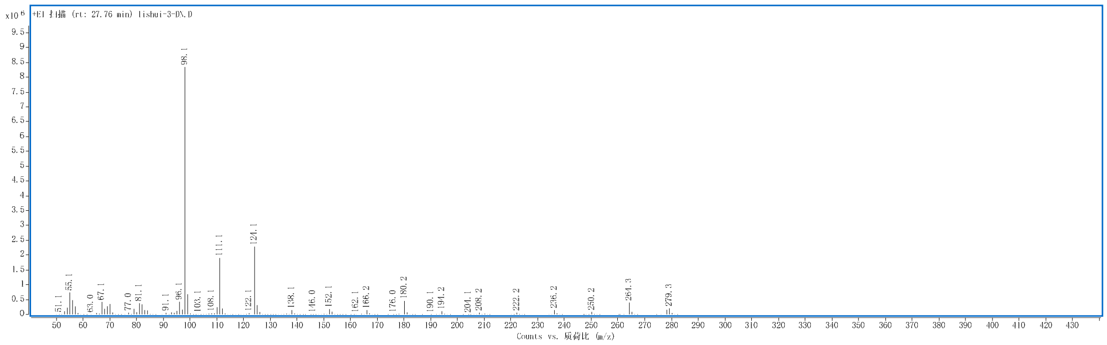

3/alkaloid

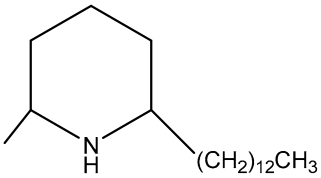

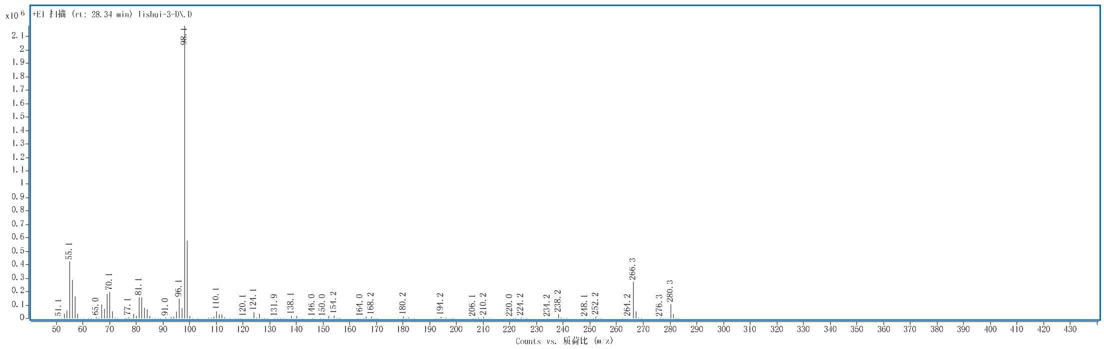

4/alkaloid

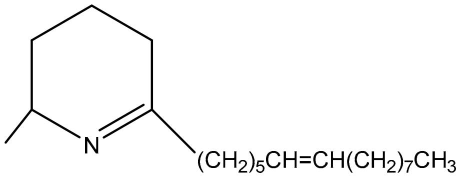

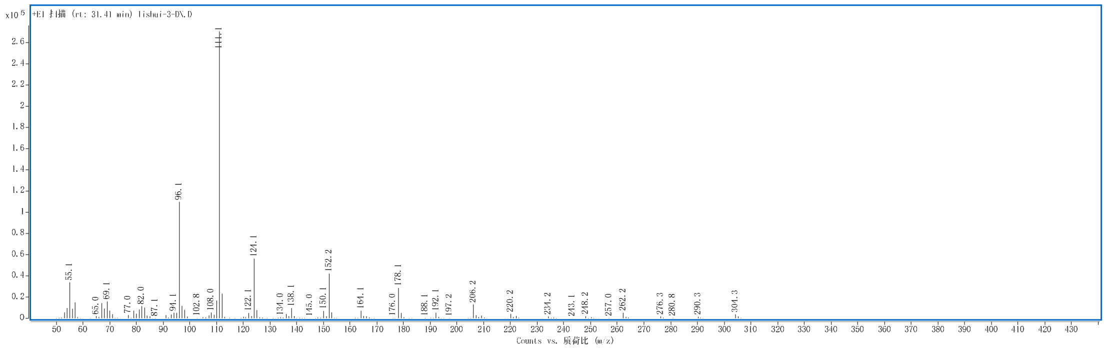

5/alkaloid

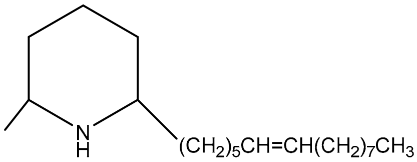

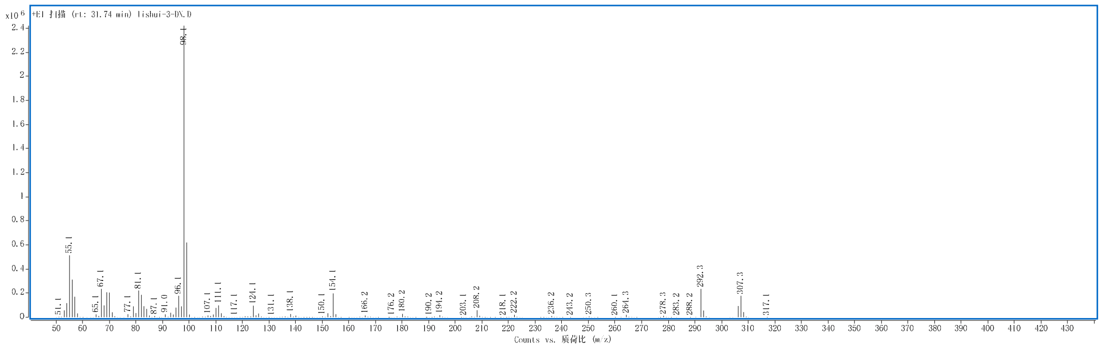

6/alkaloid

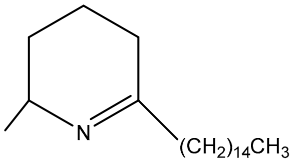

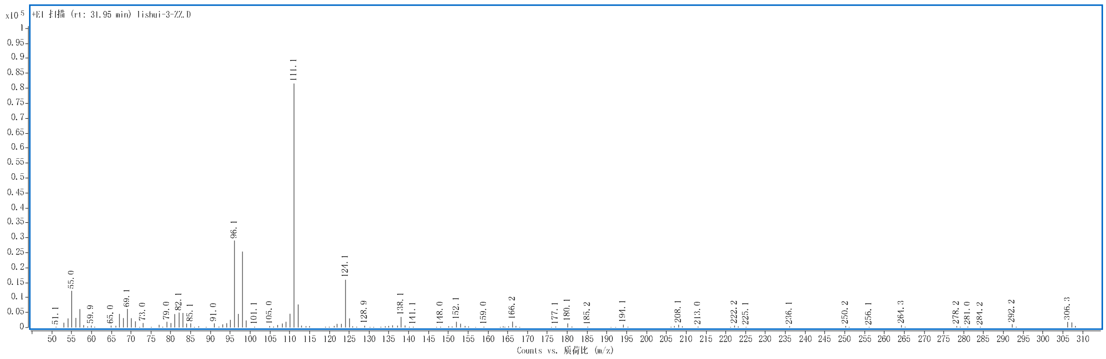

7/alkaloid

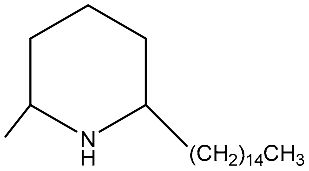

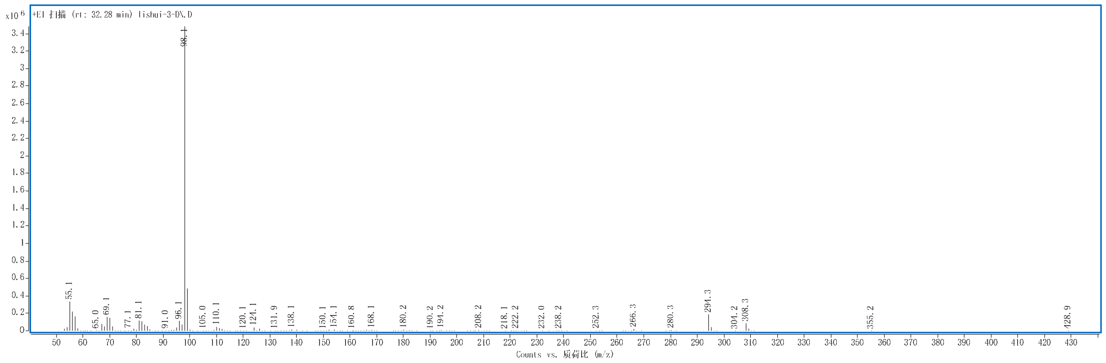

8/alkaloid

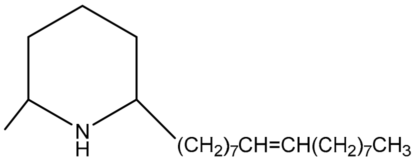

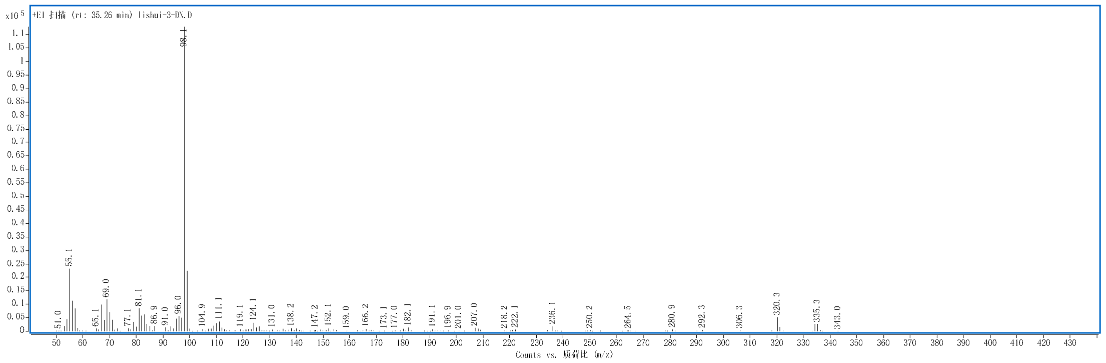

Supplement: Supplementary file 1 [file ijerph-20-01904-s001.zip › Supplemental Information S1.pdf]
