# Supplementary material for: Sensitization of Guinea Pig Skin to Imported Fire Ant Alkaloids and Establishment of an Inflammatory Model
Source: Int J Environ Res Public Health. 2023 Jan 20;20(3):1904. doi: 10.3390/ijerph20031904 (PMC9914866; doi:10.3390/ijerph20031904)

Skin irritation response of guinea pigs to different intradermal injection treatments.

AI: intradermal injection of alkaloids;

BI: *S. invicta* stings and bites;

VI: vehicle control injection;

SI: normal saline injection.

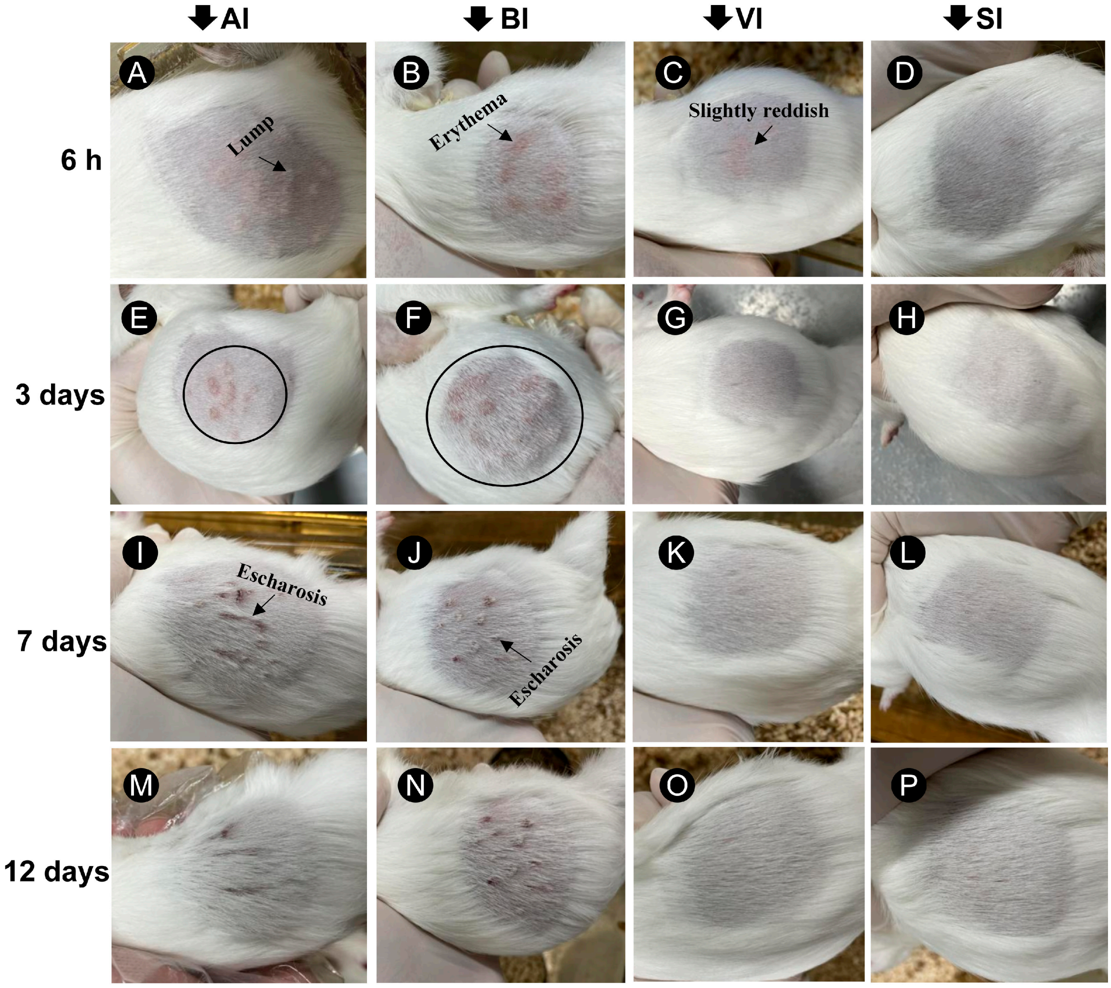

Supplement: Supplementary file 1 [file ijerph-20-01904-s001.zip › Supplemental Information S2.pdf]
